# Recent advances in the understanding and management of ARDS

**DOI:** 10.12688/f1000research.20411.1

**Published:** 2019-11-22

**Authors:** Tyler J Peck, Kathryn A Hibbert

**Affiliations:** 1 Division of Pulmonary and Critical Care, Massachusetts General Hospital and Harvard Medical School, Boston, MA, 02114, USA

**Keywords:** ARDS, acute respiratory distress syndrome, extracorporeal membrane oxygenation, prone positioning, heterogeneity of treatment effect

## Abstract

The acute respiratory distress syndrome (ARDS) remains a common and highly morbid condition despite advances in the understanding and management of this complex critical illness. Recent work has illuminated the heterogeneity within ARDS and demonstrated the likely impact of heterogeneity on the identification of effective therapeutic interventions. Despite these challenges, new data have also informed the standard of care for ARDS and have resulted in the re-evaluation of previously established therapies, including ventilation strategies, pharmacologic interventions, and rescue therapies. As the field of ARDS continues to evolve, innovative approaches will be needed to further define phenotypes within ARDS and design targeted clinical trials.

## Introduction

Since its initial definition in a case series in 1967 ^1^, there has been an ongoing evolution in the understanding and management of acute lung injury and the associated acute respiratory distress syndrome (ARDS). Despite advances in our understanding of the pathophysiologic cascade that results in ARDS, including key inflammatory mediators and disruption of the normal alveolar-capillary endothelial barrier ^2^, there remain no specific pharmacologic therapies for the condition. Instead, the interventions shown to improve outcomes in ARDS remain clinical management strategies such as lung protective mechanical ventilation and prone positioning. Overall, these interventions have improved outcomes for patients with ARDS, but the burden of lung injury remains significant with a high incidence and risk of both morbidity and mortality. Here, we will review recent advances in the understanding and management of ARDS and discuss ongoing challenges that will require further innovation.

## Acute respiratory distress syndrome: defining the syndrome and its impact

ARDS is a syndrome of respiratory failure marked by clinical features of hypoxemia and altered respiratory system mechanics. A consensus definition was refined most recently in 2012 with the Berlin definition ^3^, which features three major criteria and changed the categorization of severity. The three criteria defining ARDS are (1) onset within 1 week of known clinical insult or new or worsening respiratory symptoms; (2) bilateral opacities not fully explained by effusions, lobar/lung collapse, or nodules on chest x-ray or computed tomography; and (3) respiratory failure not fully explained by cardiac failure or fluid overload (requires objective assessment—such as echocardiography—to exclude hydrostatic edema if no ARDS risk factor is present). Other observed clinical features of ARDS include decreased lung compliance and regional heterogeneity of aeration and tissue injury. The Berlin definition also grouped patients with ARDS into categories of mild, moderate, and severe on the basis of the ratio of arterial blood partial pressure of oxygen (PaO _2_) to the fraction of inspired oxygen (FiO _2_) (P:F ratio). These categories of severity (mild: 200 < P:F ratio ≤ 300; moderate: 100 < P:F ratio ≤ 200; severe: P:F ratio ≤ 100) were applied to a cohort of over 4,000 patients gathered from clinical and physiologic trials. In this validation cohort, increasing severity corresponded well with increasing mortality. Similarly, severity of lung opacification on chest radiograph based on the Radiographic Assessment of Lung Edema (RALE) score correlated well with severity of illness and mortality as validated in the FACTT trial cohort ^4^. The Kigali modification of the Berlin definition offers alternate criteria, including peripheral capillary oxygen saturation (SpO _2_)-to-FiO _2_ ratio and chest ultrasound, which is a useful adaptation in low-resource settings ^5^.

ARDS remains a common and highly morbid condition. In the US, based on a cohort of patients studied in and around King County, Washington ^6^, the estimated annual incidence of acute lung injury is 190,600 cases and the estimated annual mortality is 74,500 patients. This corresponds to a mortality of 38.5% for patients with acute lung injury, which is similar to mortality rates seen in multiple interventional clinical trials in ARDS. More recently, a population-based cohort study ^7^ evaluated trends in ARDS incidence over the course of 8 years. Notably, the incidence of ARDS on admission remained stable, but the incidence of hospital-acquired ARDS fell significantly over the study period, suggesting that changes in care have been effective in preventing cases of iatrogenic ARDS. Additional US studies and study of the global incidence and outcomes of ARDS have shown similar numbers, including an intensive care unit incidence of 10.4% and an unadjusted mortality of 35.3% ^8, 9^. Recent study of a single-center cohort of patients in Rwanda, a lower-resource setting compared with prior studies, revealed an incidence of 4% among all hospital admissions with mortality of 50%; affected patients were younger and ARDS was more frequently associated with trauma compared with the King County cohort ^5^. A secondary analysis of the LUNG SAFE cohort compared ARDS populations between high- and middle-income countries, showing that adjusted in-hospital mortality was higher in the middle-income cohort and that lower gross national product was associated with poorer hospital survival in patients with ARDS ^10^. Overall, these data demonstrate the ongoing burden of ARDS around the world—despite recent advances—and ongoing disparities in outcomes. There is also increasing recognition of significant sequelae in ARDS survivors, including persistent functional deficits and neurocognitive morbidity such as cognitive deficits and post-traumatic stress disorder ^11^.

## Advances in understanding of acute respiratory distress syndrome

The Berlin definition of ARDS uses easily determined clinical parameters for diagnostic criteria and is purposefully inclusive of a heterogeneous cohort with varied etiologies of lung injury and diverse underlying disease states. One benefit of this definition is that patients with acute lung injury are easily identified in the clinical setting, thereby allowing both early intervention and pragmatic clinical trial design with clear enrollment criteria. However, the widely encompassing definition results in a heterogeneous population that necessarily includes patients with both different prognoses and divergent response to specific therapies. The likely impact of this heterogeneity is increasingly recognized, particularly on the background of so many failed clinical trials in ARDS and other critical illness. Although a syndromic definition allows clinical trial enrollment, a study may underestimate the impact of an intervention if there exists significant heterogeneity within the enrolled population. Specifically, a study could have an overall negative result while a small group of high-risk patients actually benefits from the intervention. Conversely, a study could show a benefit to treatment while a small subgroup of patients is actually harmed in the interventional group. If these subgroups are not easily identified, a randomized clinical trial may miss the significance of a therapy. Additionally, if enrollment in two trials includes populations that differ in the representation of these subgroups, they may have conflicting results. Further understanding of the heterogeneity within ARDS and the distinct subgroups within the syndrome is therefore important not only for interpreting completed trials but also for guiding future investigation.

The heterogeneity of ARDS is highlighted by the diversity of underlying pathologic findings on autopsy of patients with ARDS diagnosed by the Berlin criteria ^12^. Only a limited proportion of patients with clinical ARDS had diffuse alveolar damage, the histopathologic hallmark of ARDS. Many other post-mortem diagnoses, including pneumonia, pulmonary edema, cancer infiltration, pulmonary hemorrhage, and even some cases with no pulmonary lesion identified, were made in this cohort. Since biopsies are only infrequently obtained in these patients, there are a number of other methods to identify and define subgroups of patients with ARDS.

One strategy is to categorize patients by nature of insult. Acute lung injury corresponding with ARDS can be triggered by a variety of insults, including sepsis, pneumonia, trauma, pancreatitis, and toxic inhalation. These can be more broadly categorized into direct insults to the lung (for example, pneumonia and aspiration) and indirect insults to the lung (for example, sepsis and pancreatitis); these categories have been shown in retrospective studies to correlate with prognosis ^13^. ARDS can also be categorized by radiographic appearance, and some studies have correlated a diffuse versus focal pattern of disease with other biomarkers such as inflammatory mediators ^14^. Lastly, a more complex approach focuses on mechanistic difference in ARDS pathobiology, which may represent various endotypes with distinct disease processes. This approach may delineate subgroups that would respond differently to various interventions and further study of these could lead to pathway-targeted therapies.

Although ARDS endotypes have yet to be fully uncovered, recent studies have shown that patients with ARDS can be categorized into subphenotypes based on clinical features and that these subphenotypes can have differing responses to various interventions. One latent class analysis ^15^ identified two subphenotypes (a hyperinflammatory group and a hypoinflammatory group) in whom positive end-expiratory pressure (PEEP) interventions had different impacts on outcomes, including mortality, ventilator-free days, and organ failure-free days. In a similar post-hoc analysis of the HARP-2 trial ^16^, which showed no benefit with simvastatin therapy in either ventilator-free days or mortality, the hyperinflammatory subphenotype had a mortality benefit when patients received simvastatin versus placebo. This hyperinflammatory subphenotype was identified by using latent subclass analysis and was defined by several features, including higher values of soluble tumor necrosis factor receptor 1 (sTNFr1) and interleukin-6 (IL-6), lower platelet counts, and more vasopressor use compared with the hypoinflammatory subphenotype group. A post-hoc analysis of the FACCT trial cohort ^17^ identified a hyperinflammatory subphenotype (with higher serum levels of IL-8 and TNFr1 and lower serum bicarbonate levels) in which mortality was lower with the fluid-liberal strategy compared with the fluid-conservative strategy. This is notably different from the primary analysis of these data, which showed no mortality difference between the study arms. Together, these studies demonstrate the importance of understanding subgroups within ARDS in order to explore prior study data and also to inform future study design to identify effective therapies.

## Advances in management of acute respiratory distress syndrome

### Initial ventilator settings

With the lack of effective pharmacologic interventions, initial management has focused on ventilator strategies that stabilize gas exchange while avoiding further injury to lung tissue (ventilator-associated lung injury). Despite many studies on ventilator strategies and guidelines published by various professional societies to promote evidence-based management ^18, 19^, there remains a diversity of opinion on optimal management. A foundational study of ARDS demonstrated that mortality was reduced in ARDS when patients received lower tidal volumes (initial tidal volume of 6 mL/kg) compared with higher tidal volumes (initial tidal volume 12 of mL/kg) ^20^. This is theorized to be due to a reduction in overdistention of ventilated lung with high regional volumes and pressures—so-called volutrauma and barotrauma—and has become the standard of care. Subsequent studies have shown that the use of an “open lung” ventilation strategy improves lung mechanics, oxygenation, and inflammatory markers ^21^. This strategy relies on the use of optimal lung recruitment to increase the fraction of lung that is aerated with the goal of delivering the set tidal volume to the largest functional lung. Different strategies of titrating PEEP have been studied extensively. PEEP is thought to benefit patients with ARDS by preventing derecruitment of collapsible lung units at the end of exhalation, thus preventing the cyclic opening and closing of alveoli that can promote lung injury via shear stress. However, the ALVEOLI trial ^22^ previously demonstrated no benefit of high PEEP over low PEEP in ARDS when low tidal volumes and limitations of plateau pressure (PPlat) were used. More recent studies may help clarify the effect of various open lung ventilation and PEEP titration methods on outcomes.

### Driving pressure and positive end-expiratory pressure titration

Given the prior association of high airway pressures with poor outcomes in ARDS, conventional management focused on minimizing the PPlat. However, a recent retrospective analysis of patients enrolled in ARDS trials showed that the physiologic parameter best associated with outcomes was, in fact, driving pressure (PPlat – PEEP) ^23^. This variable is determined by both the compliance of the respiratory system and the delivered tidal volume and may be better associated with outcomes because in some patients a high PEEP is required to optimize recruitment (for example, in obesity), which in turn results in a high PPlat (>30 cm H _2_O) but these patients are not experiencing significant regional overdistention. Conversely, patients may be on very low PEEP and have an “acceptable” PPlat but have a very high distending pressure, reflecting a poorly compliant respiratory system and high regional strain. Data on driving pressure remain limited; it has not yet been the target of a randomized clinical trial. Additionally, in the recent ART trial ^24^ (discussed below), the intervention group had a lower mean driving pressure and higher mortality, suggesting that its predictive value is limited. However, as we await further clinical trial data, the physiologic rationale of optimizing compliance through recruitment and judicious tidal volumes to avoid overdistention, thereby minimizing driving pressure, remains strong.

The ART trial ^24^ assessed the effect of a lung recruitment maneuver and PEEP titration to the best respiratory system compliance (versus an empirical PEEP strategy) on all-cause mortality. In that study, patients in the intervention group underwent a “maximum alveolar recruitment maneuver” followed by a decremental PEEP trial to determine the level at which respiratory system compliance was optimized. Of note, the recruitment maneuver protocol was modified partway through the study after reported serious adverse outcomes, including cardiac arrest. The study authors found a significantly increased 28-day mortality in the intervention group compared with control (55.3% versus 49.3%), raising questions about the safety of such recruitment maneuvers as part of an open lung ventilation strategy. Additionally, the patients in the study cohort did not appear to be very recruitable; although driving pressure was improved in the intervention group, it was not different enough to be expected to significantly affect mortality. It remains unknown whether recruitment maneuvers with lower (and possibly safer) airway pressures in a more recruitable cohort of patients may provide the benefit of alveolar recruitment with less risk. It also remains unknown whether a decremental, physiologically targeted PEEP titration method may have benefit when not combined with such aggressive recruitment.

The placement of an esophageal catheter to measure esophageal pressure (a surrogate for pleural pressure) has also been used to guide PEEP titration. This is based on the concept that excessive transpulmonary pressure (airway pressure minus pleural pressure, the distending pressure of the lung parenchyma) is a more accurate indicator of ventilator-induced lung injury than use of airway pressure alone. However, the recent EPVent-2 study ^25^ showed no significant improvement in ARDS outcomes with use of esophageal manometry to target a combination of transpulmonary pressure and FiO _2_ compared with PEEP titration based on an empirical high PEEP-FiO _2_ table. Significantly, there was no difference in the primary outcome measure, a composite score of death and days free from mechanical ventilation through day 28. There was also no significant difference in other physiologic variables—including PEEP, driving pressure, and PPlat—between the two groups. There are therefore no data to suggest that routine use of esophageal manometry, in combination with oxygen targets, is necessary to optimize ventilator settings and minimize ventilator-associated lung injury in patients with ARDS. However, clinically, not all physicians use a combination of transpulmonary pressure and oxygen targets in titrating PEEP with an esophageal balloon, which means that this study may not reflect common practice. Additionally, esophageal balloons allow distinction of the two mechanical elements of the respiratory system (chest wall and lung parenchyma), which remains useful in complex clinical situations.

### Prone positioning

Placing patients with ARDS in the prone position has multiple physiologic effects that are potentially beneficial. It has been known for quite some time that the prone position improves oxygenation; this occurs because of both more homogeneous distribution of perfusion to the lung and recruitment of collapsed lung units, which reduces that amount of perfusion that is functional shunt. Additionally, recruitment alone may be beneficial since it increases the functional size of the lung, thereby reducing the risk of volutrauma/barotrauma, and may decrease the risk of atelectrauma. However, clinical trial data on prone positioning in ARDS have been conflicting, and there have been ongoing concerns about potential adverse events, including dislodgement of indwelling lines and the endotracheal tube and pressure ulcers ^26– 28^. A large randomized clinical trial, the PROSEVA trial ^29^, did show an impressive mortality benefit (32.8% versus 16.0% 28-day mortality) in patients who are placed in the prone position early in illness (<48 hours after ARDS onset) and who are maintained in the prone position for most of the day and until gas exchange is significantly improved. Additionally, this study did not show any increased risk for adverse events in patents placed in the prone position despite being conducted across many trial sites. These data should strongly encourage clinicians to consider prone positioning in patients with moderate to severe ARDS, and prone positioning should be considered as an upfront therapy and not a rescue therapy. However, the comparison group in the PROSEVA trial received relatively low levels of PEEP, which may have biased toward a benefit with prone position since PEEP can provide some of the same physiologic benefits as prone positioning. Therefore, a second large study comparing prone position with higher levels of—or personalized—PEEP would be useful in assessing the necessity of prone positioning in patients with ARDS.

### Pharmacologic therapy

Neuromuscular blockade (NMB) has been a common intervention for patients with moderate to severe ARDS after a randomized trial demonstrated a mortality benefit to NMB for 48 hours in patients with a P:F ratio of less than 150 early in their illness ^30^. There are multiple plausible mechanisms for the benefit of NMB in ARDS, including elimination of patient-ventilator asynchrony, which reduces the risk of volutrauma and barotrauma, and a reduction in end-expiratory derecruitment due to expiratory effort, which thereby reduces atelectrauma. These potential benefits are mirrored by a reduction in circulating inflammatory mediators in patients receiving NMB, which may also be due in part to a direct effect of NMB. However, a recent randomized trial that attempted to replicate the prior positive result showed no benefit to NMB in a similar patient cohort ^31^. Additionally, the group of patients receiving NMB were less mobile and had a greater incidence of serious adverse cardiovascular events. One criticism of that study has been that many patients were excluded because they were already receiving NMB and this may have biased against finding a benefit. Given the current body of evidence, it is reasonable to use NMB in patients in whom ventilator synchrony cannot otherwise be achieved, but there are no data to support routine use in patients with moderate to severe ARDS.

Many other pharmacologic therapies have been studied and have failed to demonstrate a clinical benefit. These include surfactant ^32^, *N*-acetylcysteine ^33^, and sivelestat ^34^ (a neutrophil elastase inhibitor). Even inhaled nitric oxide, which can provide transient improvement in oxygenation, has been repeatedly demonstrated to offer no outcome benefit and to increase the incidence of adverse renal outcomes ^35, 36^. Despite the lack of effective drug interventions thus far, there remains significant interest in identifying medications that may improve ARDS outcomes either by modifying the inflammatory process or by promoting the re-establishment of functional lung tissue.

The previously referenced HARP-2 trial ^37^ evaluated high-dose simvastatin (80 mg) compared with placebo but found no difference in either ventilator-free days or mortality at 28 days. Subgroup analyses did not identify any separate group that may benefit, including patients with sepsis or with elevated C-reactive protein levels. However, as noted above, a more recent post-hoc re-evaluation of the HARP-2 dataset has used latent class analysis to identify a subgroup characterized by a more inflammatory phenotype of ARDS that may benefit from statin therapy.

Inhaled beta-agonist therapy has been of interest as a treatment for ARDS after the observation in animal models that beta-agonist therapy enhanced alveolar fluid clearance. The ALTA trial ^38^ randomly assigned patients to receive aerosolized albuterol versus placebo but was stopped early for futility with no observed benefit in ventilator-free days at 28 days or mortality at days 60 and 90. In an attempt to gauge whether beta-agonist therapy may reduce inflammation, serum IL-6 and IL-8 levels were measured; however, there was no significant difference found between the two groups in the serum concentration of either of these cytokines. Intravenous delivery of beta-agonist therapy showed some promise of reduced pulmonary edema in early studies. However, the BALTI-2 trial ^39^, which evaluated intravenous salbutamol versus placebo, was terminated early after data showed increased 28-day mortality in the intervention group. There are therefore no data to support the use of either inhaled or intravenous beta-agonist therapy in patients with ARDS.

Perhaps the most studied and discussed pharmacologic intervention for ARDS is steroids. Multiple prior studies have shown mixed results. For example, the LaSRS trial ^40^ found that patients with ARDS for at least 7 days had a similar rate of mortality when given methylprednisolone compared with those receiving placebo. However, those patients who received methylprednisolone at least 14 days after the onset of ARDS had an increased rate of mortality compared with the placebo group, suggesting a detrimental effect of steroids in late ARDS. A more recent meta-analysis ^41^ that included the LaSRS trial found that patients receiving steroids (either methylprednisolone or hydrocortisone) had a higher rate of achieving unassisted breathing by day 28 (80% versus 50%) and had a lower rate of hospital mortality (20% versus 33%) compared with those receiving placebo. A 2019 Cochrane Review on this topic concludes that steroids may reduce 3-month mortality, but the certainty of the evidence is low ^42^. While it is possible (and perhaps likely) that a subgroup of patients with ARDS will benefit from steroids, we do not yet know which patients these are. Additionally, given the heterogeneity of the underlying pathology in patients with diagnosed ARDS, it remains possible that those patients who respond, in fact, have an undiagnosed inflammatory lung disease such as organizing pneumonia. At present, steroids are not recommended for routine use in ARDS and further study is needed.

### Rescue therapies: extracorporeal membrane oxygenation

Since its development decades ago, extracorporeal membrane oxygenation (ECMO) has been an appealing rescue therapy for patients with severe hypoxemia. Though historically more commonly employed in neonates, the use of veno-venous ECMO in adults with hypoxemic respiratory failure has been on the rise ^43^. This is due in part to the lack of other specific therapies and in part to very promising case reports demonstrating fairly low mortality in patients with severe ARDS who had not responded to other interventions ^44^. However, few randomized trials have evaluated the use of ECMO in the management of ARDS and the ones that have done so do not demonstrate a convincing benefit. The CESAR trial ^45^ randomly assigned patients with severe ARDS to either standard of care at the institution to which they presented or transfer to a specialty center for ECMO therapy. Although there was a significant difference in the primary outcome, a composite endpoint of survival without significant disability, not all patients who were randomly assigned to ECMO actually received the therapy and the patients in the “usual care” arm did not uniformly receive what is now considered standard of care, including low tidal volume ventilation. Therefore, the degree to which the improved outcomes in the intervention arm were due to ECMO alone is unclear. In order to address these ongoing questions, a large randomized trial that compared true standard of care with early ECMO was recently completed. The EOLIA trial ^46^ did not find a significant difference in mortality between the two groups and was stopped early after crossing a futility boundary. Notably, the patients in the conventional management group were allowed to “cross over” and receive ECMO if they failed standard interventions, including prone positioning, paralysis, and inhaled pulmonary vasodilators. It seems unlikely that another large randomized trial of ECMO will be undertaken soon, and although early use of ECMO for all patients with severe ARDS is not supported by the data, it remains an important rescue therapy for patients who are declining despite other interventions. Patients who are likely to have the best outcomes on ECMO are those who have few other organ failures and who are early in their illness (<7 days). Local experience in ECMO cannulation and management is also likely to be an important factor in patient outcomes, and early referral to an ECMO-capable ARDS treatment center is recommended for patients with severe ARDS.

## Future directions in acute respiratory distress syndrome

Despite improved outcomes and increased understanding of ARDS, many areas remain for future investigation and innovation; only with further progress will clinicians continue to advance care for these critically ill patients. Further delineation of subphenotypes and endotypes is necessary not only to provide a more accurate prognosis to any individual but also to inform and enrich future trials. Precise classification of patients, paired with an understanding of their underlying disease process, may increase the likelihood of identifying beneficial therapies and make real the potential of precision medicine. This may require the identification of novel biomarkers and biologic pathways but could also leverage already-available biomarkers and clinical data, as recent latent class analyses have. Development of easily-available testing of these biomarkers would make classification of patients by subphenotype more clinically feasible ^47^. The recent identification of populations who have benefitted from prior interventions highlights the likely impact of heterogeneity of treatment effect and signifies the importance of this work in advancing the science of ARDS. Ongoing studies of novel therapy, including medications ^48– 50^ and stem cell therapy ^51– 53^, and replication of prior successful studies of interventions such as prone positioning will also contribute significantly to the field. Additionally, increased understanding of optimal supportive care, including sedation targets and implementation of early mobility, is necessary to improve outcomes in these patients. Lastly, a better understanding of how to prevent ARDS and identify those at most risk may reduce the incidence of this highly morbid disease going forward.
